# Wear Behaviors of AISI 4145H Drilling Tool Steel under Drilling Fluid Environment Conditions

**DOI:** 10.3390/ma15031221

**Published:** 2022-02-06

**Authors:** Changsheng Yue, Hong Cai, Lingrong Kong, Chenfan Liang, Zhijian Peng, Yu Wang

**Affiliations:** 1 Central Research Institute of Building and Construction, MCC Group, Co., Ltd., Beijing 100088, China; yuecs163@163.com; 2 Key Laboratory on Deep GeoDrilling Technology of the Ministry of Natural Resources, School of Engineering and Technology, China University of Geosciences, Beijing 100083, China; 2002180063@cugb.edu.cn (H.C.); wangyu203@cugb.edu.cn (Y.W.); 3 School of Science, China University of Geosciences, Beijing 100083, China; pengzhijian@cugb.edu.cn

**Keywords:** wear behavior, AISI 4145H steel, abrasive wear, ASTM B611

## Abstract

4145H steel is a commonly used material for downhole tools. However, up to now the wear behavior of 4145H drilling tool steel under real drilling fluid environment conditions is still not clear. In this work, this was investigated using a modified ASTM B611 rubber ring wet grinding test system, in which six kinds of abrasives (talc, dolomite or fluorite, as well as their mixed abrasive with quartz) with metal hardness-to-abrasive hardness ratios (*H/H_A_*) ranging from 0.25 to 6.25 were used in the drilling fluid for experiments. The results show that the *H/H_A_* value determined the wear mechanism of 4145H steel. When a single soft abrasive was used (with *H/H_A_* higher than 1.3–1.5), polishing was the dominantly observed mechanism. While mixed abrasives were applied, a microcutting mechanism due to the ploughing of hard abrasive particles on the steel surface was also observed. The increase in mass fraction of the soft abrasives has little effect on the wear rate of 4145H steel, but its wear rate will significantly increase as the mass fraction of hard abrasives increases. Therefore, in order to extend the life of drilling tools and reduce downhole accidents, the mass fraction of hard particles in the drilling fluid should be reduced as much as possible.

## 1. Introduction

With the increase of drilling depth in oil and gas exploration, the failure of downhole drilling tools due to the wear, corrosion and erosion by drilling fluid has become a challenge that cannot be ignored [1,2], as they will shorten the service life of drilling tools and increase drilling costs [3,4,5,6]. Among them, one of the main ways of wear is caused by abrasive wear against solid particles contained in the drilling fluid [7,8,9,10]. Therefore, investigating the wear mechanism of drilling tool steel exposed to various particles in the drilling fluid is essential to predict the life of the tools.

During real drilling operations, the solid particles in the mud mainly include talc, dolomite, fluorite and quartz [11,12,13]. The hardness of the first three particles is lower (*H_A_* < 210), while that of quartz is greater than 1000. Therefore, the working environment of the drilling tools is an abrasive one made up of a mixture of particles of different hardness.

In the study on the relationship between abrasive hardness and wear mechanism, Tabor [14] firstly verified that when there is a hardness difference of about 20% between adjacent minerals the “ability to scratch” will occur. Khruschov [15] established a correlation between the wear resistance and physical properties of materials by abrasive wear tests against fixed abrasive grains on technically pure metals, heat-treated steels, cold work hardened materials, hard wear-resistant materials and minerals. Gundlach and Parks [16] studied the influence of abrasive hardness on the wear resistance of high chromium cast irons, indicating that their wear rate increased with increasing hardness of the abrasive. Kato [17] studied experimentally the abrasive wear mechanism of metals, reporting that the microstructure of metals caused by abrasive wear is not only related to the hardness of the abrasive, but also to the strain rate of the metals. Zum Gahr [18] found that in addition to the hardness of abrasive particles, the capability of deformation or the fracture toughness of the wearing material is also very important for the wear mechanism. Torrance [19] theoretically improved the mechanics models of abrasive process, providing a means of linking the different effects of various parameters together to determine the wear mechanism of the workpiece under particular, complex conditions. Mello et al. [20] used SiO_2_ fine abrasives to conduct free ball micro-abrasion tests and analyzed the effect of micro-structural parameters (eutectic carbides and matrix hardness) on the micro-abrasive wear of multi-component ferrous alloys abraded by soft, fine abrasive particles, revealing that the wear coefficient was neither influenced by the nature and amount of eutectic carbides nor by the hardness of the matrix, and the removal of matrix material due to abrasion with fine silica particles would decrease the mechanical supporting of the carbides, which caused a gradual fracture and their pull-out. Pintaude [21] tested two approaches using elastic-plastic models and three tribological pairs with similar ratios of abrasive hardness to the worn material hardness to gain an understanding of the hardness differential required for abrasion, theoretically confirming the empirical result that the abrasive must be at least 1.2 times harder than the worn surface if it is to produce a scratch. Pintaude et al. [22] also applied a pin-on-disk test apparatus to investigate the abrasive wear behavior of 1070 and 52,100 steels as well as ductile and white cast irons sliding with glass or alumina as the abrasive materials, revealing that the microcutting mechanism of wear prevailed together with friction coefficients larger than 0.4 when the relatively hard abrasive alumina was used; while the relatively soft abrasive glass was tested, indentation of abrasive particles followed by its fragmentation, and a creation of a thin deformed layer were the main damage mechanisms, with the friction coefficient lying below 0.4. Moreover, Piazzetta et al. [23] proposed a new approach based on scanning electron microscopy to identify the wear mechanisms acting on the Cerchar stylus (AISI A2 tool steel) during tests, indicating that the wear mechanisms could be classified into two extremes (mild and severe) with a transition region between them. The mild regime could be characterized by the polishing action of rocks (Cerchar abrasiveness index ≤ 1.8). The severe regime could be characterized by micro-cutting with extensive plastic deformation (Cerchar abrasiveness index ≥ 3.1). However, all these studies were carried out under dry conditions. 

More recently, Rong et al. [24] studied the relationship between the abrasive wear behavior of hard metal YG8B and the concentration of coarse abrasive SiO_2_, Al_2_O_3_ and SiC in their slurry, indicating that the volume loss of hard metals was positively correlated with the hardness of abrasives, their concentration in slurry and the duration of testing under the same condition, respectively, but the wear rate was positively correlated with the hardness of abrasives and their concentration in slurry, which changed in different ways with sliding distance while different abrasive slurries were used. Ren et al. [25,26] studied the wear behaviors of TiCN cermets as well as ultra-fine WC-Co hard metal RX8UF in slurries with the coarse-angle abrasives of silicon carbide, alumina and silica through using a modified ASTM B611 wet sand rubber edge wheel, revealing that the wear rate increased with increasing abrasive hardness and their mass fraction. With extending sliding distance, under lower abrasive fraction, the wear rate increased very slowly, but under higher abrasive fraction it initially increased rapidly, then became steady and even dropped down. Cai et al. [27] used a modified ASTM B611 rubber ring wet grinding test system to study the wear behavior of 4145H steel in a slurry containing SiO_2_, Al_2_O_3_ and SiC, respectively, showing that both the wear volume and wear rate of the steel increased with the concentration of abrasive particles. However, Cai et al. only selected abrasive particles with much higher hardness than that of the steel and ignored the effect of a large number of soft abrasive particles (such as talc, dolomite and fluorite) contained in the real drilling slurry.

Therefore, the purpose of this study was to simulate the wear behavior of 4145H drilling tool steel in abrasive slurrys through laboratory experiments to explore the performance of drilling tools during oil and gas drilling, so as to find the ways to reduce the wear of drilling tools. On the basis of our previous work [27], a modified ASTM B611 wet sand rubber rimmed wheel test system was used to conduct the tests on 4145H drilling steel in water-based slurry with different abrasive hardness. The wear rate and mechanism were investigated systematically by measuring the wear loss of the samples and examining the surface morphology and microstructure of the abraded 4145H steel under different experimental conditions, hoping that the wear and anti-wear performance of the drilling tools in actual working conditions could be reasonably estimated. This study might provide a guide for reducing the wear of 4145H steel drilling tools in actual engineering, extending the service life of drilling tool and reducing downhole accidents.

## 2. Materials and Methods

### 2.1. Materials

4145H steel (Hebei Lisen Petroleum Machinery Co., Ltd., Cangzhou, China) with high strength and excellent comprehensive mechanical properties was selected for the experiment, which is an international general oil drilling tool steel certified by the American Iron and Steel Institute (AISI) [27,28,29]. In order to avoid the interference of sample quality on the test, the samples used in the present tests are all mature industrial products with surface roughness *Ra* ≤ 2.733 μm and the sample quality of the same batch is controllable. The dimension of the specimens is 25.5 × 57 × 8 mm^3^ and the chemical composition of the specimens is shown in Table 1.

In this study, three kinds of low hardness abrasives (talc, dolomite, and fluorite) and quartz sand with greater hardness were used as the basic abrasives. The particle size distribution (μm), the spike parameter quadratic fitting value (SPQ) and the ratio of metal hardness-to-abrasive hardness (*H/H_A_*) are displayed in Table 2. 

The slurries used in this study consist of two basic components: abrasive and drilling fluid. In addition to 1000 mL of water, it also contains 1 wt.% high-viscosity carboxymethyl cellulose (HV-CMC), 1 wt.% xanthan gum (XG), and 5 wt.% bentonites [26]. The carboxymethyl cellulose aqueous solution has the properties of thickening, film-forming, bonding, water retention, colloid protection, emulsification, suspension, and so on. Xanthan gum is generally used as an emulsifier, stabilizer, gel thickener, sizing agent, and film-forming agent. Adding these two materials (HV-CMC and XG) in the slurry is to prevent the possible sedimentation of the abrasive particles due to the larger specific gravity so that the distribution of the abrasive particles in the slurry is more uniform.

In addition to using the three kinds of abrasives with lower hardness for independent experiments, in this study, talc, dolomite and fluorite, respectively, were mixed with quartz in a mass ratio of 1:1 as the abrasive, and then three groups of experiments with these mixed abrasives were carried out. The mass fractions of the different abrasives together with other components in the slurry are shown in Table 3. In each batch of slurry, the mass fraction of abrasive is 5%, 10%, 20%, 30% and 40%, respectively, and the total mass of drilling fluid is 1070 g. Resultantly, a total of 30 sets of tests were conducted in this study.

### 2.2. Test System

**Figure 1:**
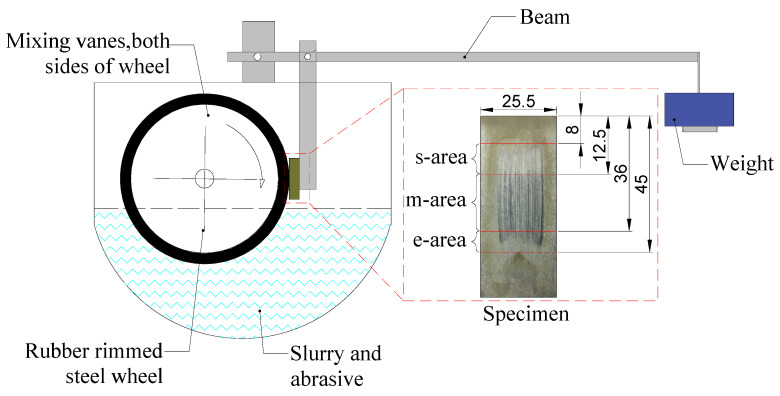
The schematic diagram of the modified ASTM B611 test system.

As a standardized test equipment, the ASTM B611 steel wheel abrasion test system has some unavoidable limitations in application. For example, the results will be influenced by the harder steel wheel when a softer abrasive was used. In order to meet the requirements of the present work, the ASTM B611 steel wheel abrasion test system was modified with a rubber wheel with a Shore hardness of 70 HA to take the place of the steel wheel in this study. The improved test system adopted in the present work is shown in Figure 1.

### 2.3. Test Procedure and Conditions

Before the test, 1000 g of water, 50 g of bentonite and the abrasives of the corresponding mass were firstly evenly mixed with a high-speed stirrer (Shandong Meike Instrument Co., Ltd., Qingdao, China). Then, 10 g XG and 10 g carboxymethyl cellulose were gradually added in the mixture during the stirring process to make the slurry gradually become uniform, ensuring that the abrasive particles were suspended in the slurry without sedimentation. Using an ultrasonic cleaning machine (Jinan Kelin Automation Equipment Co., Ltd., Jinan, China), the testing samples were washed. After cleaning, the sample was mounted onto the improved B611 equipment. Then, the prepared slurry was added into the tank, which is ready for the test.

To avoid any differences due to the large roughness of the machined surface of the samples, all the samples were pre-ground before the formal test. Through trial and error, it was found that a pre-grinding step for 8 min at a speed of 150 r∙min^−1^ (with a linear velocity of 1.415 m∙s^−1^ for the wheel) can make the average wear rate relatively stable for all the tests. Therefore, all the samples were pre-ground for 1200 r before formal tests. The formal test is divided into two stages, as shown in Table 4. In the first stage, the test machine rotates at 150 r∙min^−1^, and the weight of the sample was measured every 1200 r, for a total of 10 measurements. In the second stage, the weight of the sample was measured every 2400 r, which was also performed for 10 times, and the other experimental parameters were similar with those for the first stage experiment. The total test time for each sample was 248 min. After completing the two stages of the experiment, the tested samples were characterized except for the weight of the samples.

### 2.4. Sample Characterization

#### 2.4.1. Wear Rate

Wear rate is defined as the volume loss of the specimen per kilometer of sliding distance, which can be expressed as:

(1) $$\alpha_{i}=\frac{W_{i}}{L_{i}},$$

where $\alpha_{i}$ is the wear rate of the sample in the *i*th segment, mm^3^/km; and $L_{i}$ is the sliding distance in the *i*th segment, km. The sliding distance is the length of the testing rubber wheel sliding on the surface of the testing sample, which can be expressed as:

(2) $$L_{i}=\frac{\pi dRt_{i}}{1000},$$

where, d is the diameter of the rubber wheel, mm; R is the rotation speed of testing machine, r/min; and $t_{i}$ is the testing time of the *i-*th segment, min.

#### 2.4.2. Morphology, Surface Roughness and Microstructure

The surface morphologies of the specimens were observed by using a Laser scanning confocal microscope (3D, LEXT OLS 4100, Olympus, Tokyo, Japan). For each sample, the photos on three different areas of the sample surface during grinding were taken (divided as shown in Figure 1): the starting area (s-area), the middle area (m-area), and the end area (e-area) during the grinding process. The surface roughness of the tested sample was measured by the straight-line method, which is represented by the symbol *Ra*. The microstructure of the samples was examined by scanning electron microscopy (SEM, FEI Quanta 200 FEG, Philadelphia, PA, USA) on their worn surfaces.

## 3. Results and Discussion

### 3.1. Wear Rate by Soft Abrasives

**Figure 2:**
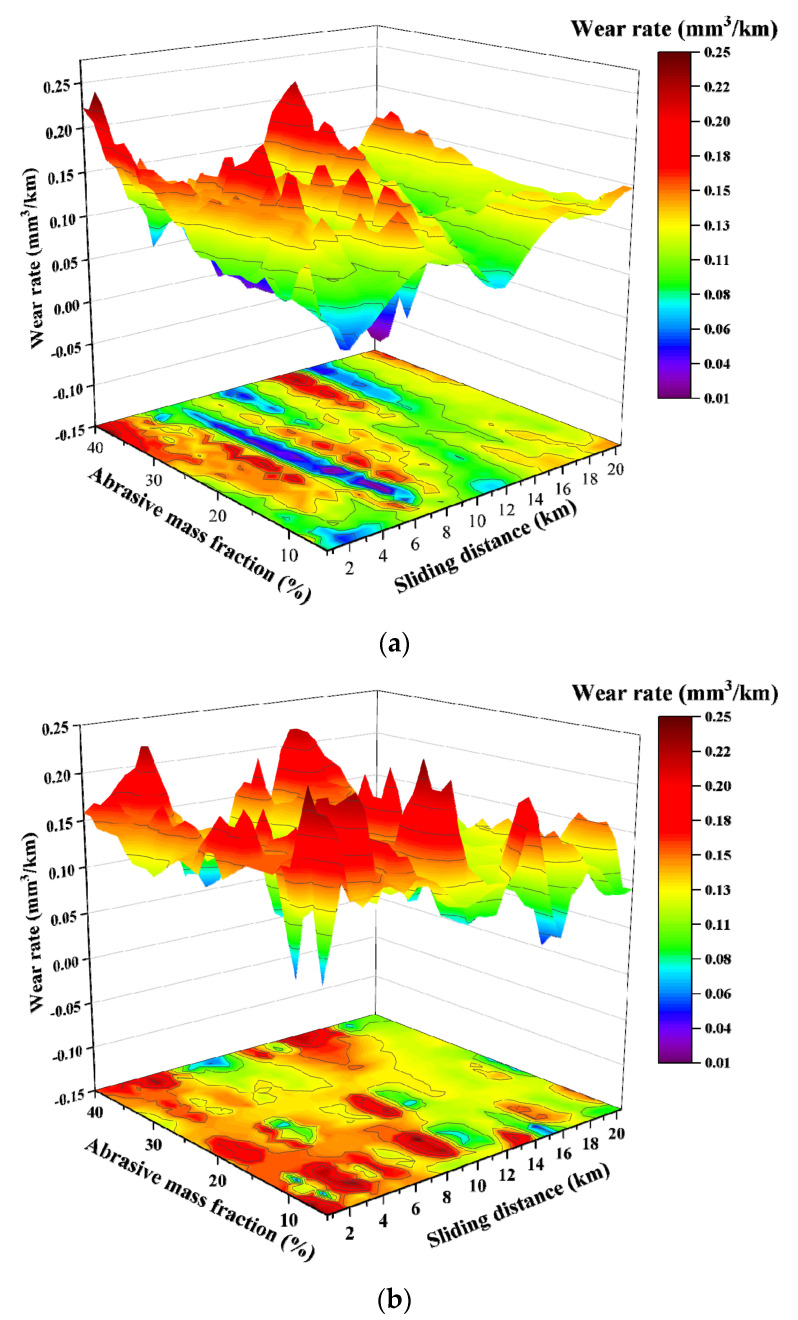

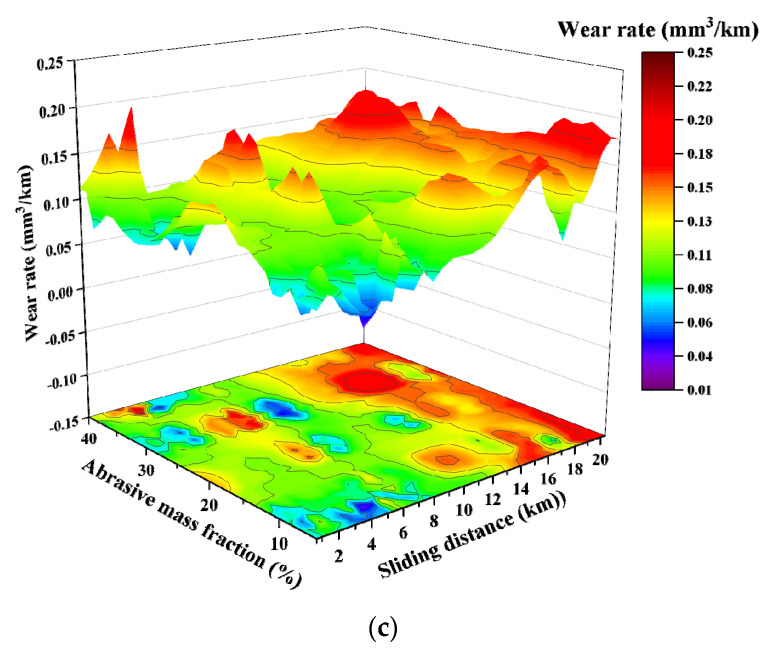
The relationship between the wear rate and sliding distance for the test groups with single soft abrasive: (**a**) talc; (**b**) dolomite; (**c**) fluorite.

The relationship between the wear rate and the relative sliding distance when using talc, dolomite and fluorite as abrasives, respectively, is shown in Figure 2. It can be seen that when the same kind of abrasive is used, as the sliding distance extends, the wear rate of the specimen does not change significantly with the increase in the abrasive mass fraction. Comparing the three groups of graphs, the wear rates of the samples are all concentrated in the range of 0.05–0.2. In addition, there are obvious irregular oscillations, which have no clear correspondence with the type and mass fraction of the abrasives.

### 3.2. Wear Rate by Mixed Abrasives

**Figure 3:**
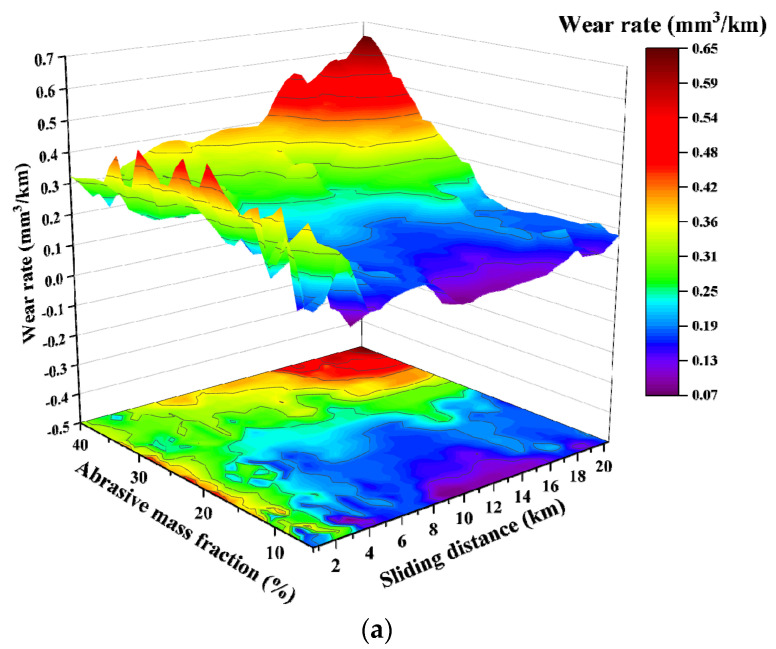

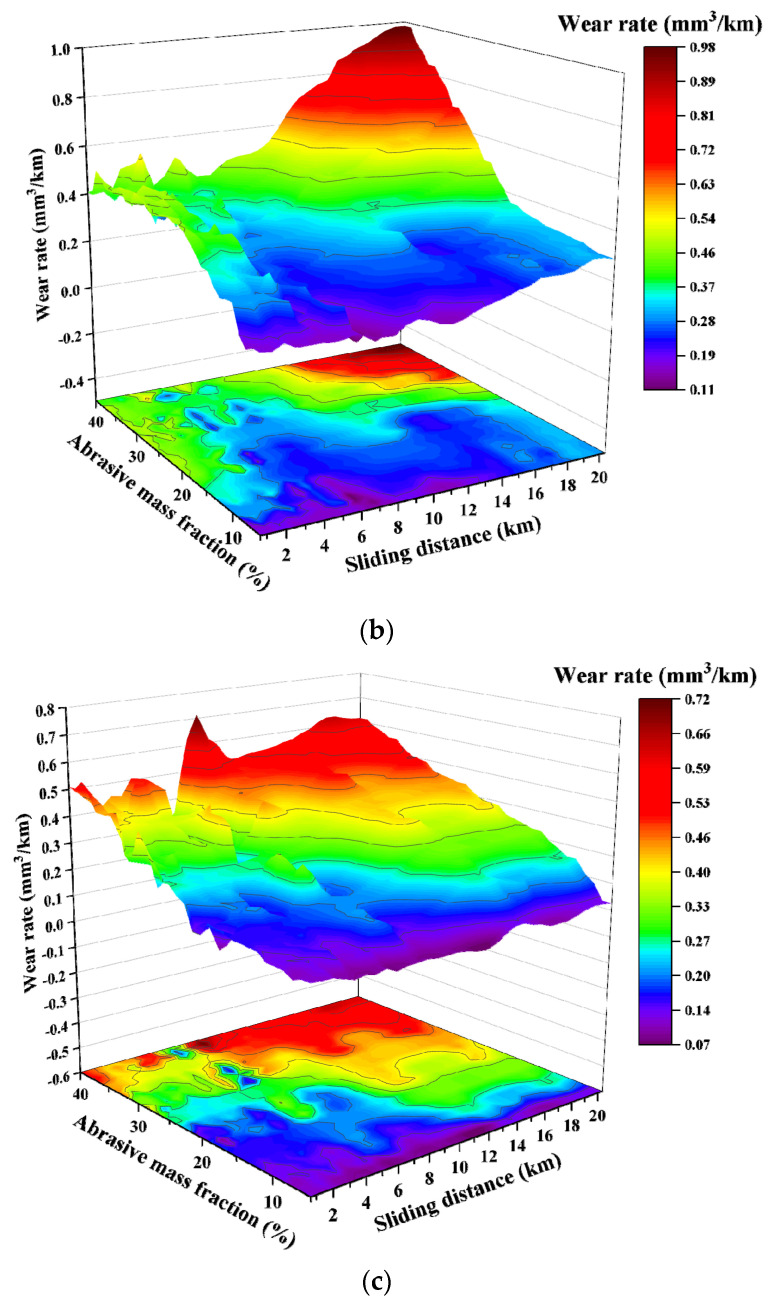
The relationship between the wear rate and the sliding distance for the test groups with mixed abrasives: (**a**) talc and quartz; (**b**) dolomite and quartz; (**c**) fluorite and quartz.

The resulting relationship between wear rate and the relative sliding distance when the softer talc, dolomite and fluorite, respectively, were mixed with the harder quartz sand in a mass ratio of 1:1 as the abrasive, is shown in Figure 3. As is seen in this figure, when a mixture of talc and quartz is used as the abrasive, with the increase in sliding distance, the recorded wear rate in the early stage of the experiment basically remains unchanged. And after the sliding distance exceeds over 10 km, the wear rate increases significantly. 

However, when a mixture of dolomite and quartz was used the abrasive, the wear rate is relatively stable at the beginning as the sliding distance increases. When the sliding distance exceeds 10 km, the value of the wear rate still increases slightly. But the numerical change of the wear rate becomes very significant when more than 30 wt.% of the abrasive mixture is used. Using a mixture of fluorite and quartz as the abrasive, the value of the wear rate increases slightly, but the overall stability can be kept as the sliding distance increases.

### 3.3. Morphology and Microstructure of the Abraded Surfaces

#### 3.3.1. Three-Dimensional Morphology by Softer Abrasives

**Figure 4:**
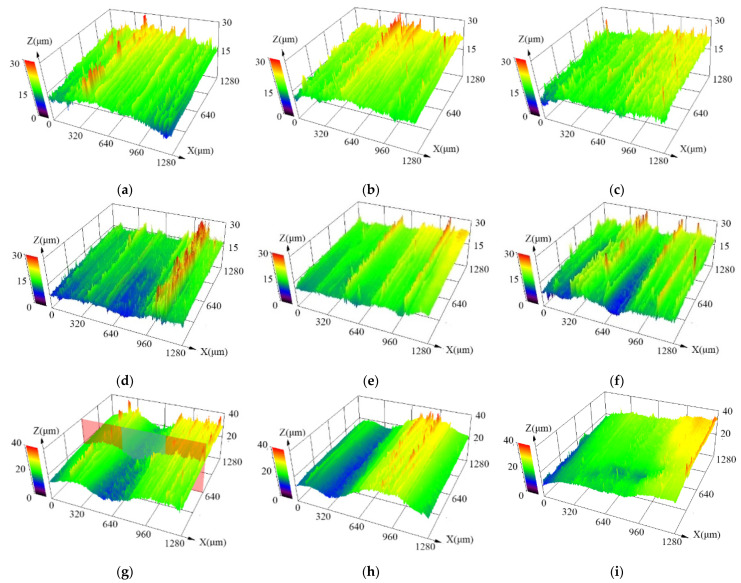
Three-dimensional surface morphology in the middle area of the AISI 4145H steel after being abraded by slurries with different softer abrasives: talc (**a**–**c**), dolomite (**d**–**f**) and fluorite (**g**–**i**). The slurries in (**a**,**d**,**g**) contains 5 wt.%, (**b**,**e**,**h**) 20 wt.%, and (**c**,**f**,**i**) 40 wt.% of abrasives.

Talc has the lowest hardness among the three soft abrasives, and the hardness ratio of 4145H steel to talc (*H/H_A_*) is 5–6.25 (see Table 2). Figure 4a–c shows the three-dimensional surface morphology in the middle area of the specimens after the abrasion tests with slurries containing different mass fractions of talc. The measured surface roughness of typical samples is presented in Table 5. As is seen, although there are lots of scratches of quite high depth on the sample surface, the surface of the samples is generally smooth with a quite low roughness of little fluctuation. The roughness of the abraded surfaces is much lower than that of the pristine surface for all the samples in this group, indicating a polishing effect on them. With increasing mass fraction of talc in the slurry, the roughness of the abraded surface decreased, revealing a better polishing effect caused by the slurry with talc. Moreover, the surface roughness of the starting area, the middle area and the end area for each sample decreases in turn, which could be attributed to the different load that the different area bears [27].

The hardness of dolomite is slightly larger than that of talc, and the hardness ratio of 4145H steel to dolomite (*H/H_A_*) is 1.5–1.9 (see Table 2). Figure 4d–f exhibits the three-dimensional surface morphology in the middle area of the specimens after the abrasion tests using slurries containing different mass fractions of dolomite. The roughness of the sample surface is presented in Table 6. Similar with the case using talc as the abrasive, although there are also some scratches of quite high depth on the sample surface, the surface of the samples is smooth with a quite low roughness of little fluctuation. The roughness of the abraded surfaces is also much lower than that of pristine surface, indicating a polishing effect still exists during the test with the slurry of dolomite. With increasing concentration of dolomite in the slurry, the roughness in the starting area of the abraded surfaces increases significantly, while those in the middle area and the end area increase first and then decrease. As a result, the roughness in certain areas of the abraded surfaces is somewhat higher than that abraded by talc, implying a microcutting effect on the steel surface caused by the harder abrasive dolomite. Moreover, the surface roughness of the starting area, the middle area and the end area for each sample also decreases in turn, which could be attributed to the different loads they bear [27]. 

Fluorite has the largest hardness among the present three soft abrasives, and the hardness ratio of 4145H steel to fluorite (*H/H_A_*) reaches 1.3–1.5 (see Table 2). Figure 4g–i displays the three-dimensional surface morphology in the middle area of the specimens after the abrasion tests using slurries containing different mass fractions of fluorite. The roughness of the sample surface is exhibited in Table 7. As is seen, compared with those of the other two groups of samples with talc or dolomite, although the abraded surfaces are generally very smooth, there were some scratches of very high depth on the sample surface. As a result, the abraded samples tested with fluorite generally present the largest average surface roughness among the samples with the three soft abrasives under the same conditions, although they are still lower than that of the pristine surface, revealing a significant polishing effect with more serious microcutting effect on the samples after tests with the slurries containing fluorite abrasive. With increasing concentration of fluorite in the slurry, the roughness in the middle area of the abraded surfaces decreases, while those in the starting area and the end area increase first and then decrease, revealing that the fluorite abrasive presents a stronger microcutting effect on the surface of 4145H steel. Similar with the previously two groups of samples with talc or dolomite, the surface roughness of the starting area, the middle area and the end area for each sample with fluorite also decreases in turn.

#### 3.3.2. Three-Dimensional Morphology by Mixed Abrasives

**Figure 5:**
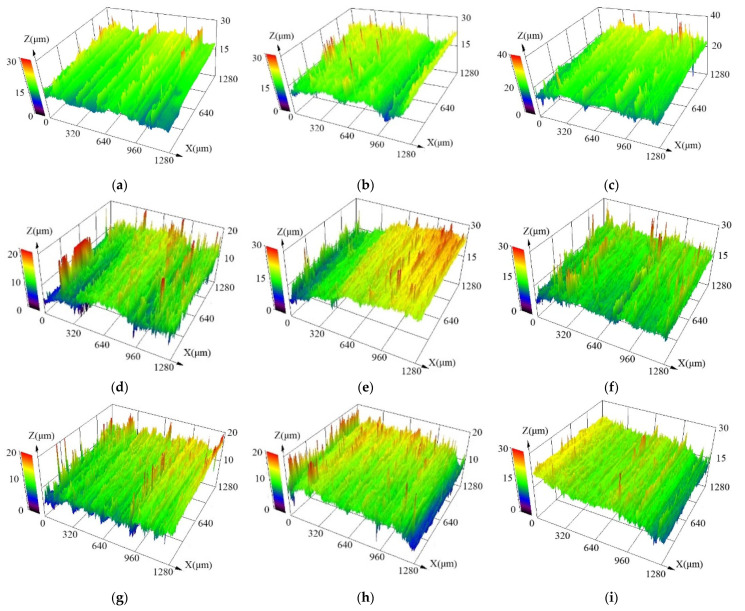
Three-dimensional surface morphology in the middle area of AISI 4145H steel after being abraded by the slurries with mixed abrasives: talc and quartz (**a**–**c**), dolomite and quartz (**d**–**f**), and fluorite and quartz (**g**–**i**). The slurries in (**a**,**d**,**g**) contains 5 wt.%, (**b**,**e**,**h**) 20 wt.%, and (**c**,**f**,**i**) 40 wt.% of the mixed abrasives.

Figure 5a–c shows the three-dimensional surface morphology in the middle area of the specimens after the wear tests with slurries containing different mass fractions of the mixed abrasives of talc and quartz. The measured surface roughness of the samples is shown in Table 8. Compared with the results mentioned in Section 3.3.1, the surface roughness of the specimens in this group is generally larger than those of the samples tested with slurries of single soft talc abrasive, confirming the higher microcutting effect of the hard quartz abrasive. However, their roughness is still smaller than that of the pristine sample, implying the polishing effect still exists. With increasing mass fraction of the mixed abrasives talc and quartz, the surface roughness of the tested samples first increases and then decreases. For each sample, the surface roughness in the starting area, the middle area and the end area basically decreases first and then increase. 

Figure 5d–f exhibits the three-dimensional morphology in the middle area of the specimens after the wear tests with slurries containing different mass fractions of the mixed abrasives of dolomite and quartz. The measured surface roughness of the abraded samples is presented in Table 9. As is seen, compared with the case with talc and quartz, the surface roughness of the present samples with dolomite and quartz increased sharply, and the values of the surface roughness in this group of samples are all greater than 10 μm, much higher than that of the pristine samples, revealing a strong microcutting effect for the samples. As the mass fraction of the mixed abrasives in the slurries increases, the roughness for all areas on the sample surfaces generally decreases. Moreover, when the abrasive mass fraction is 5 wt.%, the surface roughness of the sample decreases in turn from the starting area, the middle area, to the end area. However, when the abrasive mass fraction is more than 20 wt.%, the surface roughness of the sample increases in turn from the starting area, the middle area, to the end area. 

Figure 5g–i displays the three-dimensional morphology in the middle area of the samples after the wear tests with slurries containing different mass fractions of mixed abrasives of fluorite and quartz. The surface roughness of the abraded samples is shown in Table 10. As is seen, this group of samples presented the largest value of surface roughness among all the samples abraded by the slurries with mixed abrasives, indicating the strongest microcutting effect for the present samples, which can be also confirmed by the furrow as shown in Figure 5g–i. With increasing mass fraction of the mixed abrasives, the surface roughness for all areas of the abraded samples first decreases very little, and then increases significantly. For each sample, the surface roughness in the starting area, the middle area and the end area first decreases significantly and then increases slightly in turn. 

### 3.4. Wear Mechanism

In this study, because different areas on the 4145H steel sample surface are subjected to different forces, their wear behavior will be different. In the starting area for wearing, a small horizontal force is applied. Due to the sudden loading, the trajectory of the abrasive particles contacting with the sample surface is unstable. Unstable abrasive particles will lead to scratches in multiple directions on the surface of the sample [27], generally resulting a higher surface roughness for this area (see Section 3.3). In the middle area, the load between the sample and the rubber wheel can ensure that the position of the abrasive particles is relatively stable. Therefore, the scratches formed on this area of the abraded sample surface are relatively uniform (also see Section 3.3).

**Figure 6:**
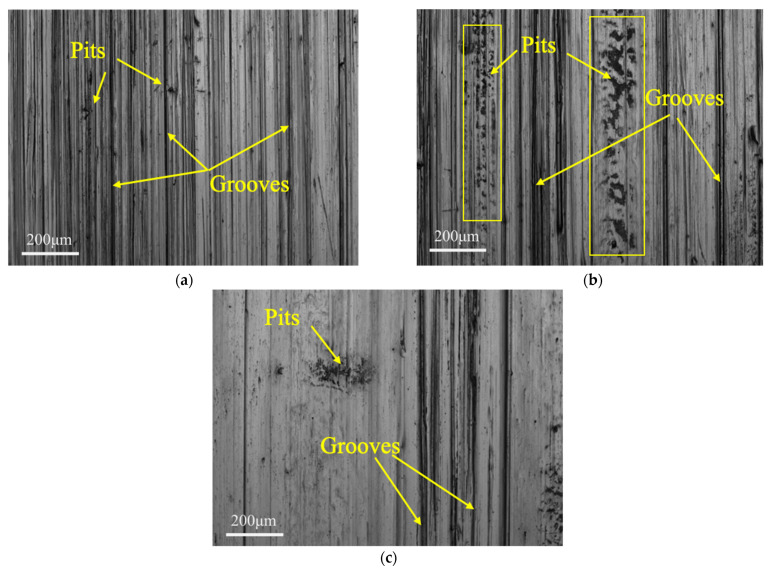
SEM images in the middle area on the sample surfaces after the wear test with slurries containing 20 wt.% of a soft abrasive: (**a**) talc, (**b**) dolomite and (**c**) fluorite.

Figure 6 shows typical SEM images in the middle area of the abraded surfaces after the tests with slurries containing 20 wt.% of a single soft abrasive (talc, dolomite and fluorite, respectively). Grooves can obviously be seen on the abraded surfaces, which are generally formed from the machining of the original sample surfaces. After the tests with slurries of a softer abrasive (talc or dolomite), more grooves were left on the sample surface although the depth of the grooves might be relatively shallow (see Figure 6a,b, and also refer to Figure 4). As a result, low surface roughness was measured for them. When the harder fluorite abrasive was applied, the depth of the grooves was still very deep (see Figure 6c) although their amount was obviously reduced, indicating a stronger microcutting effect on the sample surface. Except for the grooves, however, the other parts on the same surface were quite smooth, revealing that a stronger polishing effect on the surface of the samples still happened during the tests with the soft abrasive fluorite. As a result, the samples will still have a relatively small surface roughness. These results are basically consistent with the observation by optical microscopy presented in Section 3.3.1. In addition, there are many pits on the surface of the samples, which might be formed by the tearing-off of the steel from the main body due to the fatigue wear of steel when the soft abrasive particles continuously squeeze the surface of the sample under the load for a long time [27]. 

**Figure 7:**
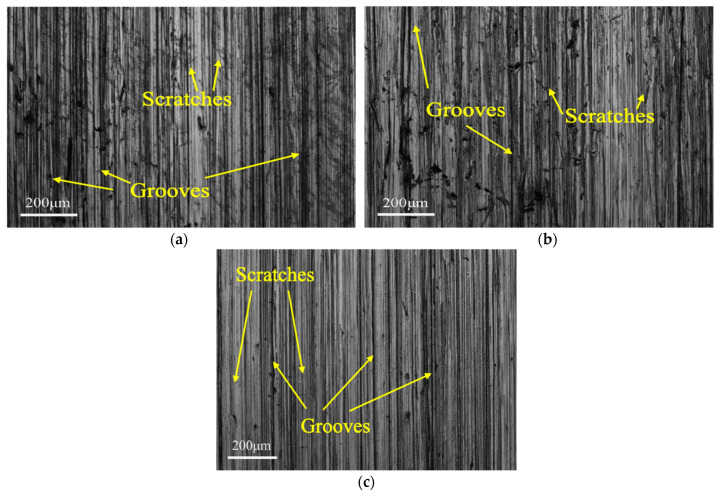
SEM images in the middle area on the sample surfaces after the wear test with slurries containing 20 wt.% of a mixed abrasive: (**a**) talc and quartz, (**b**) dolomite and quartz, and (**c**) fluorite and quartz.

Figure 7 shows typical SEM images in the middle area of the abraded surfaces after the tests with slurries containing 20 wt.% of the mixed abrasives (talc and quartz, dolomite and quartz, as well as fluorite and quartz, respectively). As can be seen, more but shallower grooves were formed along the sliding direction on the abraded surface of the samples, compared with those produced during the tests with single soft abrasive. These grooves were formed by the interaction of the original machining and the microcutting of the hard abrasive particles. This result reveals that the micro-cutting effect of the abrasive is enhanced and the polishing effect is weakened after the addition of hard abrasive particles. As a result, these samples will have higher surface roughness although the maximum depth of grooves may decrease (see Section 3.3.2). Meanwhile, many irregular scratches can be observed on the surface of the samples, which are caused by the micro-cutting of quartz particles while they are squeezed out during the tests. When the mixed abrasive particles interact with the sample, the soft abrasive particles (talc, dolomite and fluorite) roll on the surface of the sample, while the hard abrasive quartz particles plow through the surface of the sample. Therefore, micro-cutting and polishing are both presented as the main mechanism [27,30].

In a word, the abrasive hardness has a great impact on the wear mechanism of 4145H steel. When a single soft abrasive was used (with *H/H_A_* higher than 1.3–1.5), the abrasive particles will only be squeezed to the surface of the sample and polishing is the main wear mechanism [22,23]. While the mixed abrasives were applied, the surface of 4145H steel is severely worn out, mainly due to the micro-cutting of quartz particles on the steel surface. It is characteristic of rapid material loss, rough sample surface, and formation of metal wear debris [27,30]. 

## 4. Conclusions

The wear behavior of AISI 4145H drilling tool steel under real drilling fluid environment conditions was evaluated by a modified ASTM B611 wet sand rubber wheel test system using slurries containing different abrasives (talc, dolomite or fluorite, as well as their mixtures with quartz) and mass fractions (5%, 10%, 20%, 30%, and 40%). The wear loss, wear rate, surface morphology, surface microstructure and wear mechanism under different testing conditions are compared. The following conclusions can be drawn:

(1) The metal hardness-to-abrasive hardness ratio (*H/H_A_*) dominates the wear mechanism of 4145H steel. When a single soft abrasive was used (with *H/H_A_* higher than 1.3–1.5), polishing ias the observed main mechanism. When mixed abrasives were applied, a microcutting mechanism due to the ploughing of hard abrasive particles on the steel surface was also observed.
(2) The increase in mass fraction of the soft abrasives in the slurries has little effect on the wear rate of 4145H steel. However, with increasing mass fraction of hard abrasive and extending sliding distance, the wear rate of 4145H steel will increase significantly.
(3) In order to extend the service life of the drilling tools and reduce downhole accidents, the mass fraction of hard particles in the drilling fluid should be reduced as much as possible.

## Figures and Tables

**Table 1:** Chemical composition of the applied 4145H steel in this work.

C	Si	Mn	P	S	Cr	Mo	Fe
0.42~0.49	0.15~0.35	0.65~1.15	≤0.035	≤0.035	0.75~1.20	0.15~0.25	Bal.

**Table 2:** Properties of the abrasives used in the test.

Abrasive	Particle Size Distribution (μm)			SPQ	Hardness (H_A_, V30)	*H/H_A_*
	d10 *	d50	d90			
Talc	85	384	541	–	45–56	5–6.25
Dolomite	411	605	811	–	145–180	1.5–1.9
Fluorite	310	491	699	–	180–210	1.3–1.5
Quartz	388	581	888	0.561	1000–1100	0.25–0.28

* The figure represents the diameter of the sieve through which the percentage (by mass fraction) of the abrasive particles can pass.

**Table 3:** The compositional mass of the slurry in the test [26].

Components	Mass Fraction of Abrasive			
	10%	20%	30%	40%
Abrasive (g)	52.6	111.1	250.0	428.6
HV-CMC (g)	10.0	10.0	10.0	10.0
X.G. (g)	10.0	10.0	10.0	10.0
Bentonite (g)	50.0	50.0	50.0	50.0

**Table 4:** The optimal test procedure and conditions for each specimen [13].

Parameters	Pre-Grinding	Testing Stage 1 (Contains 10 Times)	Testing Stage 2 (Contains 10 Times)
Setting of each time (r)	1200	1200	2400
Load (N)	225	225	225
Liner velocity of wheel (m·s^−1^)	1.415	1.415	1.415

**Table 5:** Roughness of AISI 4145H steel after being abraded by slurry with talc.

Abrasive	Concentration (wt.%)	Area Observed	*Ra* (μm)
Talc	5	Starting	1.27 ± 0.003
Talc	5	Middle	0.83 ± 0.005
Talc	5	End	0.78 ± 0.002
Talc	20	Starting	0.97 ± 0.003
Talc	20	Middle	0.81 ± 0.004
Talc	20	End	0.76 ± 0.002
Talc	40	Starting	0.91 ± 0.001
Talc	40	Middle	0.80 ± 0.002
Talc	40	End	0.65 ± 0.004

**Table 6:** Roughness of AISI 4145H steel after being abraded by slurry with dolomite.

Abrasive	Concentration (wt.%)	Area Observed	*Ra* (μm)
Dolomite	5	Starting	1.04 ± 0.002
Dolomite	5	Middle	0.83 ± 0.003
Dolomite	5	End	0.78 ± 0.005
Dolomite	20	Starting	1.22 ± 0.001
Dolomite	20	Middle	1.18 ± 0.003
Dolomite	20	End	1.04 ± 0.002
Dolomite	40	Starting	1.66 ± 0.003
Dolomite	40	Middle	0.98 ± 0.005
Dolomite	40	End	0.63 ± 0.006

**Table 7:** Roughness of AISI 4145H steel after being abraded by slurry with dolomite.

Abrasive	Concentration (wt.%)	Area Observed	*Ra* (μm)
Fluorite	5	Starting	1.50 ± 0.002
Fluorite	5	Middle	1.34 ± 0.001
Fluorite	5	End	1.06 ± 0.004
Fluorite	20	Starting	1.52 ± 0.002
Fluorite	20	Middle	1.27 ± 0.003
Fluorite	20	End	1.35 ± 0.005
Fluorite	40	Starting	0.83 ± 0.002
Fluorite	40	Middle	0.82 ± 0.002
Fluorite	40	End	0.56 ± 0.004

**Table 8:** Roughness of AISI 4145H steel after being abraded by slurry with the mixed abrasives of talc and quartz.

Abrasive	Concentration (wt.%)	Area Observed	*Ra* (μm)
Talc and quartz	5	Starting	1.06 ± 0.003
Talc and quartz	5	Middle	0.63 ± 0.004
Talc and quartz	5	End	0.82 ± 0.003
Talc and quartz	20	Starting	1.26 ± 0.002
Talc and quartz	20	Middle	1.05 ± 0.004
Talc and quartz	20	End	1.25 ± 0.005
Talc and quartz	40	Starting	1.25 ± 0.004
Talc and quartz	40	Middle	0.92 ± 0.002
Talc and quartz	40	End	0.80 ± 0.001

**Table 9:** Roughness of AISI 4145H steel after being abraded by slurry with the mixed abrasives of dolomite and quartz.

Abrasive	Concentration (wt.%)	Area Observed	*Ra* (μm)
Dolomite and quartz	5	Starting	18.29 ± 0.012
Dolomite and quartz	5	Middle	15.22 ± 0.027
Dolomite and quartz	5	End	15.06 ± 0.023
Dolomite and quartz	20	Starting	14.14 ± 0.031
Dolomite and quartz	20	Middle	14.99 ± 0.025
Dolomite and quartz	20	End	15.10 ± 0.029
Dolomite and quartz	40	Starting	11.84 ± 0.017
Dolomite and quartz	40	Middle	13.04 ± 0.035
Dolomite and quartz	40	End	20.96 ± 0.054

**Table 10:** The roughness of AISI 4145H steel after being abraded by slurry with the mixed abrasives of fluorite and quartz.

Abrasive	Concentration (wt.%)	Area Observed	*Ra* (μm)
Fluorite and quartz	5	Starting	16.11 ± 0.043
Fluorite and quartz	5	Middle	10.93 ± 0.031
Fluorite and quartz	5	End	11.51 ± 0.026
Fluorite and quartz	20	Starting	16.08 ± 0.038
Fluorite and quartz	20	Middle	10.74 ± 0.015
Fluorite and quartz	20	End	11.51 ± 0.035
Fluorite and quartz	40	Starting	20.49 ± 0.024
Fluorite and quartz	40	Middle	15.53 ± 0.035
Fluorite and quartz	40	End	17.72 ± 0.039

## Data Availability

Not applicable.
